# Clinical and laboratory findings in patients with anti-myelin oligodendrocyte glycoprotein antibodies: experience from two tertiary hospitals in Madrid

**DOI:** 10.3389/fimmu.2026.1809873

**Published:** 2026-05-11

**Authors:** Sara Muñoz-Gómez, Roberto Pariente-Rodríguez, Juan Luis Chico-García, Iván García de la Torre, Joaquín Navarro Caspistegui, José Manuel García-Domínguez, María Teresa Schiaffino, Paloma Sánchez-Mateos

**Affiliations:** 1 Department of Immunology, Hospital General Universitario Gregorio Marañón, Madrid, Spain; 2 Instituto de Investigación Sanitaria Gregorio Marañón (IISGM), Hospital General Universitario Gregorio Marañón, Madrid, Spain; 3 Department of Immunology, Hospital Universitario Ramón y Cajal, Madrid, Spain; 4 Instituto Ramón y Cajal de Investigación Sanitaria (IRYCIS), Hospital Universitario Ramón y Cajal, Madrid, Spain; 5 Department of Neurology, Hospital Universitario Ramón y Cajal, Madrid, Spain; 6 Department of Neurology, Hospital General Universitario Gregorio Marañón, Madrid, Spain; 7 Department of Immunology, Ophthalmology and Otolaryngology, School of Medicine, Universidad Complutense, Madrid, Spain

**Keywords:** anti-MOG antibodies, CSF analysis, MOGAD, myelin oligodendrocyte glycoprotein, OCBs, κ-FLC index

## Abstract

**Background/Objective:**

This study aimed to describe the clinical, radiographic and laboratory characteristics of a cohort of patients diagnosed with anti-myelin oligodendrocyte glycoprotein (anti-MOG) antibodies at two collaborating centers in Madrid (Spain). Besides the study of anti-MOG antibodies in serum and cerebrospinal fluid sample (CSF), this study highlights the results of the cytochemical study of CSF, kappa free light chain (κ-FLC) index and oligoclonal bands (OCBs).

**Methods:**

Records of patients diagnosed with anti-MOG antibodies at Hospital General Universitario Gregorio Marañón and Hospital Universitario Ramón y Cajal between 2018 and 2024 were retrospectively reviewed.

**Results:**

The cohort comprised fifteen patients. Of those patients, nine (60%) were female, ten (67%) were Caucasian, and four (27%) were children. The mean age at onset was 46 years (range, 1–66 years). Optic neuritis was the most common presentation, identified in eleven patients (73%). A clear positive MOG-IgG titer was identified in serum samples of ten patients; also, a positive result was found in a CSF sample of one patient. CSF analysis showed CSF-OCBs in three patients, low positive κ-FLC index in three patients, elevated protein in two patients, and pleocytosis in another two patients. Relapses were reported in seven patients (46.6% of patients), all of whom were female patients.

**Discussion:**

This study provides valuable insights into the characteristics observed in patients diagnosed with anti-MOG antibodies in Spain. Results are discussed in the context of the 2023 international diagnostic criteria for MOGAD and previously published cohort studies.

## 1 Introduction

Myelin oligodendrocyte glycoprotein antibody-associated disease (MOGAD) is an autoimmune disorder of the central nervous system (CNS) characterized by the presence of antibodies targeting the myelin oligodendrocyte glycoprotein (MOG). In 2007, MOGAD was recognized as a unique clinical entity due to its clinical, radiological and immunological characteristics that distinguish it from other demyelinating diseases (1); however, it was not until 2023 that the International MOGAD Panel proposed diagnostic criteria to standardize its identification and classification (2).

MOGAD is a demyelinating syndrome clinically distinct from multiple sclerosis (MS) and aquaporin-4 antibody-positive neuromyelitis optica spectrum disorder (AQP4-NMOSD), although there may be some clinical overlap between MOGAD and AQP4-negative NMOSD (3). MOGAD is typically associated with a variety of clinical phenotypes, including acute disseminated encephalomyelitis (ADEM), optic neuritis (ON), cortical encephalitis, transverse myelitis (TM) and brainstem syndromes (4).

MOGAD may have a monophasic or recurrent clinical course. Recent studies have associated elevated serum MOG-IgG levels with an increased risk of relapse. Patients with relapsing disease often have a history of other autoimmune diseases. This highlights the need for close monitoring and appropriate therapeutic strategies (5, 6).

The most recommended methods for the detection of MOG-IgG are cell-based assays (CBAs), either live or fixed (7). Serum testing is preferred, although cerebrospinal fluid (CSF) analysis may be considered in patients with high clinical suspicion but negative serum results, as a small percentage of patients may exhibit CSF-restricted MOG antibodies (8). Other common laboratory findings in patients with MOGAD are pleocytosis and proteinorrachia. Oligoclonal bands (OCBs) are rarely detected, which helps differentiate it from MS (9). The kappa free light chain (κ-FLC) index has been also proposed as a valid alternative to OCB testing in MS; however, its diagnostic utility in MOGAD has only recently begun to be explored (10).

The 2023 international criteria for diagnosing MOGAD emphasize the importance of clinical presentation, neuroimaging and laboratory findings, primarily the detection of MOG-IgG antibodies, for accurate identification and classification of patients (2). This observational study aims to report the clinical and laboratory findings of a cohort of patients diagnosed with MOG-IgG antibodies across two collaborating centers. In addition, the findings of this study are discussed in the context of the diagnostic criteria proposed by the 2023 international panel and compared with data previously reported in the literature.

## 2 Methods

### 2.1 Patients

We retrospectively recruited patients from Hospital General Universitario Gregorio Marañón and Hospital Universitario Ramón y Cajal. The study was conducted in accordance with the Declaration of Helsinki and approved by the Ethics Committee of Hospital General Universitario Gregorio Marañón and the Ethics Committee of Hospital Universitario Ramón y Cajal (protocol code: MOGAD_INM_2025_v1). Patients were recruited consecutively without exception if they fulfilled the inclusion criteria: a positive result for MOG-IgG in serum and/or CSF using a fixed CBA, and at least one initial or follow-up visit at one of the two collaborating centers between 2018 and 2024. Patients were excluded if their case had previously been reported in other published studies.

This study primarily reports data from the total cohort. For some comparative analysis, patients were stratified into children (age <18 years) and adults (age ≥18 years). In addition, adult patients were further categorized into early-onset MOGAD (EO-MOGAD; age at onset: 18–50 years) and late-onset MOGAD (LO-MOGAD; age at onset: ≥50 years) groups.

### 2.2 Clinical, imaging and laboratory data

Demographic, clinical, radiographic, and laboratory data were collected for each patient. Demographic and clinical characteristics included age at diagnosis, sex, ethnicity, clinical features at diagnosis, preceding and subsequent clinical manifestations of MOGAD, neurological diagnosis, number of relapses, and follow-up duration at the diagnosis center. A relapse was defined as the occurrence of a new clinical attack occurring at least 30 days after the onset of the previous one. Radiographic data were obtained from magnetic resonance imaging (MRI) studies performed in all patients during the acute clinical presentation or during follow-up. MRI examinations were conducted according to the standard protocols of each participating center, with gadolinium-based contrast agents used when clinically indicated.

Laboratory results included serum and/or CSF MOG-IgG titer, OCBs, CSF/serum albumin quotient (QAlb), IgG index, κ-FLC index, CSF pleocytosis (with CSF white cell counts >5/μL) and CSF total protein. MOG-IgG detection was performed semi-quantitatively using the commercial biochip immunofluorescence fixed CBA from Euroimmun (NMOSD Screen 1). In this assay, serum samples were tested at 1:10, 1:20 and 1:100 dilutions; whereas CSF samples were analyzed undiluted. The serum MOG-Ig titer was divided into two groups according to Banwell et al. (2): clear positive (fixed CBA result with a titer ≥1:100) and low positive (fixed CBA result with a titer ≥1:10 and <1:100).

### 2.3 Statistical analysis

Statistical analyses were performed with GraphPad Prism v10.2.2 (GraphPad Software, San Diego, California, USA). Quantitative variables are presented as mean, median and interquartile range (IQR), and categorical variables are presented as number of cases and percentage (%). Fisher’s exact test was used to compare categorical variables. A *p*-value <0.05 was considered statistically significant. Patients with missing data were excluded from the corresponding analyses.

## 3 Results

### 3.1 Demographic and clinical presentation

A total of fifteen consecutive patients identified with serum MOG-IgG were enrolled in this study, including six males and nine females (male/female = 1:1.5). The age at diagnosis ranged from 1 to 66 years (four children and eleven adults), with a median of 46 years (IQR = 13–58 years). The majority of patients were Caucasian (10/15, 66.7%). More detailed demographic characteristics are provided in **Table 1**.

**Table 1:** Demographic and clinical characteristic of the patients with MOG-IgG.

Characteristic	Value
Age at diagnosis (years), median (IQR)	46 (13–58)
Pediatric patients (n = 4)	11 (3-13)
Adult patients (n = 11)	49 (62-39)
Sex, n (%)	
Male	6 (40%)
Female	9 (60%)
Male: Female ratio	1:1.5
Ethnicity, n (%)	
Caucasian	10 (66.7%)
Latino	3 (20%)
African	1 (6.7%)
Asian	1 (6.7%)
Neurological phenotype at diagnosis, n (%)	
Optic neuritis (ON)	11 (73.3%)
Acute disseminated encephalomyelitis (ADEM)	2 (13.3%)
Myelitis	1 (6.7%)
Encephalitis with seizures	1 (6.7%)

The most common neurological diagnosis was ON, observed in eleven patients (73.3%), with similar proportions of these patients having bilateral ON (6/11, 54.5%) and unilateral ON (5/11, 45.5%). Other phenotypes included ADEM in two patients (13.3%), myelitis in one patient (6.7%) and encephalitis with seizures in one patient (6.7%).

### 3.2 MRI findings

MRIs were performed in all patients, with most examinations (14/15) conducted at the time of initial clinical presentation. Among these, imaging findings were consistent with the neurological diagnosis in eleven patients. In the remaining three patients, MRI findings at clinical onset were not compatible with the final diagnosis; however, all three showed clear positivity for MOG-IgG antibodies. Clinically, one patient presented with ON, another was diagnosed with autoimmune encephalitis with seizures and memory impairment, and the third exhibited features consistent with transverse myelitis, although only a cranial MRI performed. On the other hand, one patient underwent follow-up MRI while in remission from relapsing ON, which showed no demyelinating lesions.

### 3.3 Serum and CSF MOG-IgG titers

Serum MOG-IgG was the main inclusion criterion in the study cohort. All patients were MOG-IgG positive at 1:10 dilution. Only thirteen patients were tested at 1:100 dilution, with ten of them remaining positive (76.9%) (**Supplementary Table 1**). Laboratory findings are shown in **Table 2**.

**Table 2:** Laboratory findings of the patients with MOG-IgG.

Laboratory parameters	n/total (%)
MOG- IgG titer	
Clear positive in serum	10/13 (76.9%)
Low positive in serum	15/15 (100%)
Positive in CSF	1/8 (12.5%)
OCBs	
Type I pattern	11/14 (78.6%)
Type II pattern	1/14 (7.1%)
Type III pattern	2/14 (14.3%)
Elevated QAlb	5/14 (35.7%)
Elevated IgG index	2/14 (14.3%)
Pleocytosis (>5 white blood cells)	2/14 (14.3%)
Proteinorrachia	2/14 (14.3%)

Regarding the persistence of MOG-IgG positivity, five patients were tested at follow-up, all of whom were in remission. Serum MOG-IgG remained detectable in four of the five (80%) patients. The MOG-IgG initially detected at 1:100 dilution decreased to low titers or became undetectable. In two patients, MOG-IgG was only tested at low titers initially and remained low, making it impossible to determine whether the titers were initially higher and then decreased.

Concerning the detection of MOG-IgG in CSF, eight patients were evaluated and one of them yielded a positive result (**Table 2**). This patient initially presented with ON with no other abnormalities in CSF analyses (OCBs, QAlb, IgG index, total protein and leukocytes) and a high MOG-IgG titer in serum.

### 3.4 Other CSF biomarkers

The presence of OCBs was assessed in fourteen patients. Two patients exhibited type III OCBs (mirror pattern, with identical OCBs detected in both serum and CSF), and one patient showed type II OCBs (OCBs present exclusively in the CSF and absent in serum). The remaining patients showed type I OCBs (polyclonal response with absence of OCBs in both CSF and serum) (**Table 2**).

In the same fourteen patients of the study cohort, QAlb and IgG index analysis were performed. No abnormalities were observed in seven patients (50%). Five patients (35.7%) demonstrated an increased QAlb, indicating blood-brain barrier dysfunction, whereas two patients (14.3%) showed an elevated IgG index, suggestive of intrathecal synthesis of IgG. Regarding OCBs detection, none of the three patients with positive OCBs showed an altered IgG index; and only one of the two patients with type III OCBs exhibited an elevated QAlb.

κ-FLCs were evaluated in six patients. In three cases, the κ-FLCs index could not be calculated due to κ-FLC concentrations in CSF below the detection limit. In the other three patients, the κ-FLC index ranged from 4 to 19, with intrathecal synthesis of κ-FLC in the CSF <0.5 mg/L, low Q-Kappa on the Reibergram, and no OCBs observed in the CSF.

The study also included CSF white cell counts and total protein concentrations. CSF analysis revealed pleocytosis in two of the fourteen patients. As expected, increased total protein levels in the CSF were found in the same two patients who showed elevated QAlb. No differences were observed in demographic characteristics, clinical phenotype, recurrence rate or MOG-IgG titers between patients with normal and abnormal CSF findings.

### 3.5 Relapse and follow up period

**Figure 1:**
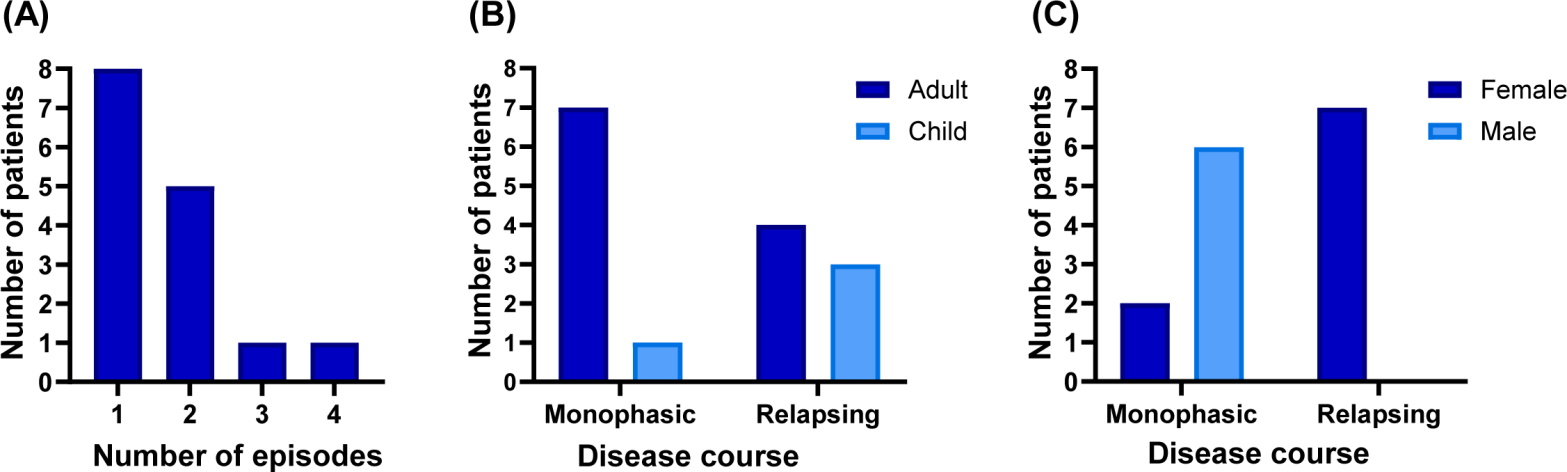
Recurrence in the study cohort. **(A)** Number of episodes per patient. **(B)** Relapse frequency according to age; relapses were more frequent in children than in adults (75% vs 36.4%) without statistical significance (Fisher’s exact test, *p* = 0.282). **(C)** Relapse frequency according to sex, with all relapse events occurring in female patients (Fisher’s exact test, *p* = 0.007).

Patients were followed for a median duration of 21 months (IQR: 8–26 months). During follow-up, seven patients experienced clinical relapses (46.6% of patients), with a median time to relapse of 6 months (IQR = 2–27 months). Number of relapses experienced since disease onset are provided in **Figure 1A**.

Despite the limited cohort size, a higher frequency of relapses was observed in children than in adults (75% of children and 36.4% of adults), although this difference was not statistically significant (*p* = 0.282) (**Figure 1B**). All reported relapses occurred in female patients while no recurrent events were observed in male patients during the follow-up period (**Figure 1C**). Patients who experienced relapses also tended to lack proteinorrachia at the time of first MOG-IgG detection, compared to 2/8 no-relapsing patients with proteinorrachia. Three of the four (75%) patients with persistent anti-MOG positivity experienced relapses, whereas the only patient whose follow-up serum sample tested negative for MOG-IgG did not have any further relapses.

### 3.6 Comparison of Early-Onset MOGAD and Late-Onset MOGAD

**Figure 2:**
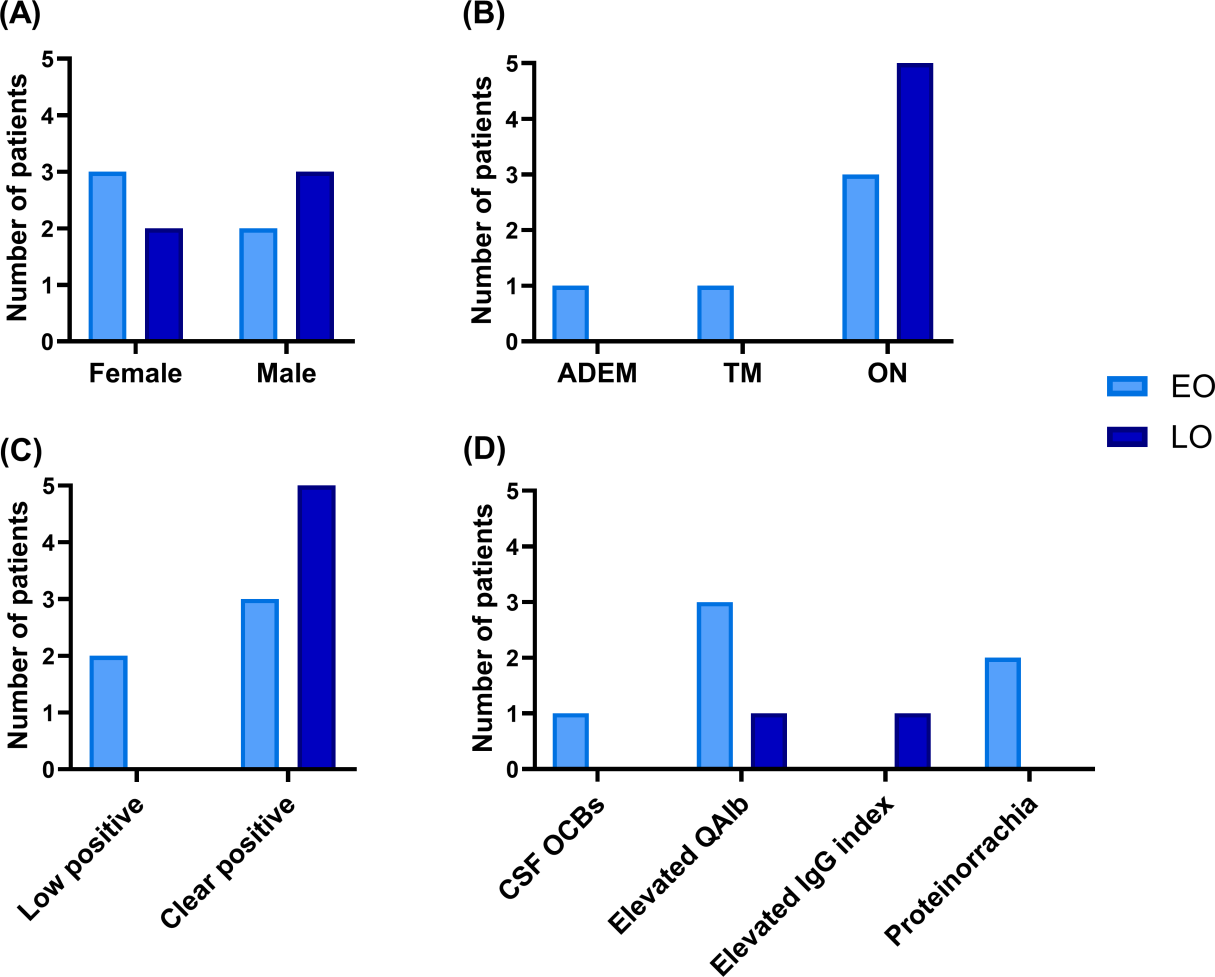
Characteristics of early-onset (EO) and late-onset (LO) MOGAD patients. **(A)** Sex. **(B)** Neurological phenotype at disease onset. **(C)** Serum MOG- IgG titer. **(D)** CSF laboratory findings. ADEM, acute disseminated encephalomyelitis; TM, transverse myelitis; ON, optic neuritis; CSF OCBs, cerebrospinal fluid oligoclonal bands; QAlb, CSF/serum albumin quotient.

Adult patients who met the 2023 MOGAD criteria (10/11) were classified by age into LO-MOGAD (n=5) and EO-MOGAD patients (n=5) (**Figure 2**). LO-MOGAD patients tended to present with several recurring characteristics: ON was the onset phenotype, MOG-IgG was detected at clear positive titer, no pleocytosis or proteinorrachia was observed and OCB analysis exhibited a type I pattern (only 4/5 analyzed). These features may represent trends within this subgroup, although they should be interpreted with caution given the limited sample size.

## 4 Discussion

This study presents a cohort of fifteen patients with anti-MOG antibodies, contributing to the expansion of current evidence on MOGAD, which remains a relatively poorly described neurological disorder. Here we provide valuable clinical and laboratory data from the experience of two tertiary centers in Madrid.

MOGAD is a relatively recently defined pathology, whose diagnostic criteria were proposed in 2023 (2). Nine of the ten patients with clear MOG-IgG positivity satisfied the main proposed criteria to be diagnosed with MOGAD: a core clinical attack and clear positive serum MOG-IgG test results measured by a CBA (2). An exception was identified in a pediatric case with clear MOG-IgG positivity who did not fulfill the clinical criteria due to presenting autoimmune encephalitis with isolated epileptic seizures without MRI abnormalities. Similarly, Zhang R et al. (11) reported an analogous pediatric case. Ramanathan et al. (12) described the presence of anti-MOG antibodies in pediatric cases who initially presented with isolated seizures with a normal MRI, but later developed typical demyelination. According to Foiadelli T et al. (13), seizures in MOGAD can occur before the first demyelinating episode (22.2% of cases). Therefore, the presence of anti-MOG antibodies in our patient may precede the demyelinating lesion observable by MRI. Furthermore, it should be noted that the clinical phenotypic spectrum of MOGAD is currently expanding, so it cannot be ruled out that this presentation represents a phenotype that has yet to be characterized. Though, after 13-months of follow-up, this patient had a second epileptic seizure in the absence of immunosuppressive treatment and continued to show no demyelinating lesions on MRI, underscoring the need for close monitoring given the potential for future demyelination.

According to international criteria, patients with low positive MOG-IgG who present with one of the main core clinical attack must also exhibit at least one supporting clinical or MRI finding in order to be diagnosed with MOGAD (2). Most of our patients with low antibodies testing had MRI findings consistent with the disease, with the exception of one patient who could not be tested for MOG-IgG at 1:100 dilution and was in remission after having experienced recurrent episodes of ON six years prior to the MRI. However, recent studies have demonstrated that MRI lesions during MOGAD attacks are highly dynamic and may disappear on follow-up imaging. In fact, Cacciaguerra et al. (14) analysed over 580 T2-lesions in MOGAD patients and observed resolution in 78% of cases, 89% of which occurred within the first 12 months. Unlike in MS or AQP4-NMOSD, MOGAD lesions often fully resolve following the acute episode, frequently leaving no detectable sequelae on conventional neuroimaging (15).

Therefore, thirteen of our fifteen patients of our cohort with MOG-IgG strictly meet the 2023 international diagnostic criteria for MOGAD. In terms of our clinical data, ON was the most frequent initial presentation in these patients, followed by ADEM, as reported in previous studies (16). Regarding demographic data, although MOGAD does not show a clear sex predominance, a slight female predominance has been observed in both previous studies (2, 16–19) and our cohort (male:female = 6:7).

Variable rates of CSF MOG antibody positivity have been shown by recent studies (20, 21). We identified one patient with serum-positive/CSF-positive MOG antibodies, and no patients with CSF-restricted MOG-IgG. The usefulness of detecting MOG-IgG in CSF for the diagnosis of MOGAD has been widely questioned. Therefore, CSF testing for MOG-IgG is recommended when there is a high level of suspicion for MOGAD in MOG-IgG seronegative patients (2, 22). Although MOG-IgG was considered to be peripherally synthesized and its presence in CSF was due to passive transfer across a disrupted blood-brain barrier (23), cases with CSF-restricted MOG-IgG suggest possible intrathecal synthesis (20, 24, 25). Further investigation is needed to clarify the role of this intrathecal production in the pathogenesis of MOGAD, including the use of experimental models.

Other tests that can be performed on CSF when MOGAD is suspected include the study of OCBs, IgG, albumin, κ-FLC index, leukocytes and total proteins. OCBs are a highly sensitive marker for MS diagnosis, but their specificity is reduced by their detection in other pathologies, including MOGAD (26). In our cohort, one patient exhibited CSF-restricted OCBs, a finding that has been described in 10-20% of MOGAD patients (16, 23, 26, 27). Additionally, two of our three patients with serum MOG-IgG and CSF-OCBs experienced relapses. While limited by the small sample size, this observation could be in line with prior reports suggesting that CSF-OCBs may be associated with an increased risk of relapse and higher IgG index values (26, 28, 29). In another regard, Jarius et al. (30) reported a higher rate of OCB positivity in patients with myelitis than in those with ON, a trend that could not be evaluated in our cohort. With regard to the κ-FLC index, its value as a biomarker for MOGAD is being questioned given the high sensitivity observed in patients with MS (31, 32). In our study, none of the patients showed an elevated κ-FLC index, which is usually observed almost exclusively in MS (10), although this observation should be specifically assessed in future studies.

Pleocytosis in CSF was detected in 8, 3% of our MOGAD patients, which is a low frequency compared to the 50% described in the literature (16, 30). Nevertheless, significant differences in pleocytosis rates have been reported between samples taken during acute attacks and remission (30), and some CSF samples in this study were collected during remission. One of the patients with pleocytosis presented with ADEM, consistent with the higher frequency reported in patients with ADEM or TM (70-80%) than in patients with ON (20-30%) (30, 33, 34).

Regarding CSF protein levels, two MOGAD patients in our cohort exhibited hyperproteinorrachia. This is a lower frequency than the 30-50% described in larger cohorts (16, 30). Although it has been proposed that proteinorrachia in the initial CSF sample doubles the risk of relapse (34), none of our patients experienced a relapse during follow-up (10 and 22 months, respectively).

Previous studies have indicated that approximately half of MOGAD patients experience a relapsing course after the initial attack (16, 35, 36), which is in line with the 38% of our MOGAD patients who developed recurrences. Several studies have reported a lower relapse rate in pediatric patients (18, 19, 37). However, in our cohort two of the three pediatric MOGAD cases experienced relapse, which tend to be a higher proportion than adults, although limited size of groups makes difficult to establish a robust conclusion. Relapse rate among female patients tends to be higher pointing to a possible sex-related predisposition to disease activity, which aligns with several prior reports (19, 38, 39). Cheng J et al. (36) suggest that sex only affects the relapse rate and not the incidence rate, and propose that these relapses may follow a different immune mechanism in males and females. Previous studies have suggested that persistent anti-MOG seropositivity may be associated with an increased risk of recurrence (19, 34). In our cohort, three of the four patients with persistent anti-MOG antibody positivity experienced a second clinical event, which is in line with this observation, although further validation in larger cohorts is required.

Finally, we explored potential differences associated with the age of onset of MOGAD in adults, classifying patients into two groups: LO-MOGAD and EO-MOGAD. Despite our limited sample size, our findings were consistent with those of previously reported larger cohorts, showing that LO-MOGAD patients more frequently presented with ON and clear positive MOG-IgG results at disease onset (40–42). Also, none of the patients exhibited hyperproteinorrachia, pleocytosis or CSF-restricted OCBs. Fan et al. (40) describe worse disability outcomes in LO-MOGAD patients, which they associate with poorer recovery in the first episode, decreased CNS tissue reserve and reduced remyelinating capacity with ageing. Dinoto et al. (41) argue that this higher frequency of cognitive decline could be related to comorbidities or aging itself. They emphasize the importance of considering MOGAD in patients aged 50 or older, as 25% in their cohort had LO-MOGAD, and up to 30% were initially misdiagnosed, affecting treatment decisions.

This study has several limitations. The small sample size and short study period restrict the statistical power, but MOGAD is a rare disease with low prevalence, which inherently limits the recruitment of large cohorts. Moreover, the use of fixed CBA may result in lower sensitivity compared to live CBA, although both methods demonstrate high specificity and high level of agreement. Additionally, as a retrospective study, not all variables were available for every patient. Notably, MOG-IgG titers were only monitored in some patients, which did not allow for further analysis of changes in antibody levels in relation to patient progress. Finally, given that the cohort was defined by MOG-IgG positivity, the inclusion of patients who do not meet the 2023 MOGAD diagnostic criteria limits the extent to which conclusions about MOGAD can be drawn.

In summary, the findings from our cohort of patients with anti-MOG antibodies highlight their heterogeneity. Most cases fulfilled current diagnostic criteria for MOGAD and showed characteristics consistent with international series, supporting MOGAD as a distinct entity within demyelinating disorders of the CNS. Nevertheless, MOG-IgG positive patients who do not meet the international diagnostic criteria emphasize the need to continue refining the current diagnostic framework.

## Data Availability

The raw data supporting the conclusions of this article will be made available by the authors, without undue reservation.
