# Supplementary material for: Clinical and laboratory findings in patients with anti-myelin oligodendrocyte glycoprotein antibodies: experience from two tertiary hospitals in Madrid
Source: Front Immunol. 2026 May 11;17:1809873. doi: 10.3389/fimmu.2026.1809873 (PMC13199094; doi:10.3389/fimmu.2026.1809873)
Supplement: Supplementary file 1 [file Table1.docx]

Supplementary Material

**SUPPLEMENTARY TABLE 1.** Case-by-case summary of clinical, radiological, and serological features supporting the diagnosis of MOGAD

| **Case** | **Clinical phenotype at onset** | **MOG-IgG Low positive dilution (≥1:10 and <1:100)** | **MOG IgG Clear positive dilution (1:100)** | **MRI compatible with clinical diagnosis** | **MRI timing** | **Meets full Banwell 2023 criteria** |
| --- | --- | --- | --- | --- | --- | --- |
| A | Bilateral optic neuritis | positive | negative | yes | at diagnosis | yes |
| B | Epileptic encephalitis | positive | positive | no | at diagnosis | no |
| C | Bilateral optic neuritis | positive | N/A | yes | at diagnosis | yes |
| D | Left optic neuritis | positive | positive | yes | at diagnosis | yes |
| E | Bilateral optic neuritis | positive | positive | yes | at diagnosis | yes |
| F | Left optic neuritis | positive | N/A | no | remission | no |
| G | Transverse myelitis | positive | positive | no | at diagnosis | yes |
| H | Bilateral optic neuritis | positive | positive | yes | at diagnosis | yes |
| I | Right optic neuritis | positive | positive | yes | at diagnosis | yes |
| J | Right optic neuritis | positive | negative | yes | at diagnosis | yes |
| K | Bilateral optic neuritis | positive | positive | yes | at diagnosis | yes |
| L | Bilateral optic neuritis | positive | positive | no | at diagnosis | yes |
| M | ADEM | positive | positive | yes | at diagnosis | yes |
| N | Right optic neuritis | positive | positive | yes | at diagnosis | yes |
| O | ADEM | positive | negative | yes | at diagnosis | yes |

N/A: not available

**
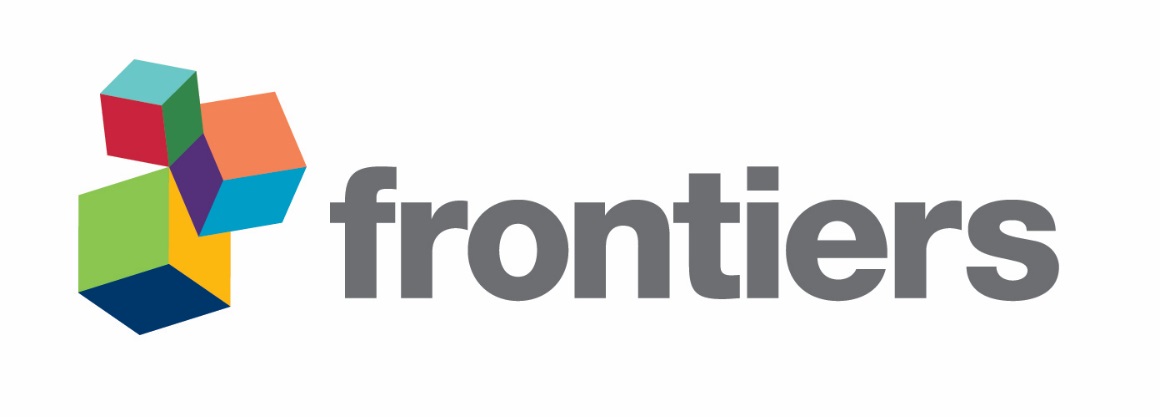
**
